# Mono-WNV and combined WNV/TBEV inactivated vaccine efficacy against a wide range of WNV and TBEV strains

**DOI:** 10.3389/fimmu.2025.1715371

**Published:** 2025-12-12

**Authors:** Ksenia Tuchynskaya, Mikhail Vorovitch, Yuriy Kruglov, Guzal Mostipanova, Ivan Kholodilov, Alla Ivanova, Victoria Kuchina, Anastasia Rogova, Galina Karganova

**Affiliations:** 1 Laboratory of Biology of Arbovirus, Federal State Autonomous Scientific Institution “Chumakov Federal Scientific Center for Research and Development of Immune-and-Biological Products of Russian Academy of Sciences” (Institute of Poliomyelitis), Moscow, Russia; 2 Department of Encephalitis Vaccine, Federal State Autonomous Scientific Institution Chumakov Federal Scientific Center for Research and Development of Immune-and-Biological Products of Russian Academy of Sciences (Institute of Poliomyelitis), Moscow, Russia; 3 Institute of Translational Medicine and Biotechnology, Sechenov First Moscow State Medical University, Moscow, Russia

**Keywords:** WNV, TBEV, inactivated vaccine, combined vaccine, orthoflaviviruses, flaviviruses

## Abstract

West Nile virus (WNV) is widespread throughout the world. Occasionally, it causes outbreaks of the West Nile fever (WNF) disease, which can lead to severe CNS damage or death. At the same time, the virus’ expanding range is increasingly leading to the formation of mixed foci with other orthoflaviviruses, such as tick-borne encephalitis virus (TBEV). Based on long-term experience using inactivated vaccines to target tick-borne encephalitis (TBE), it seems sensible to create a complex inactivated vaccine targeting two antigens to protect the population against several orthoflaviviruses at once. The immunogenicity and efficacy of the mono-WNV and combined WNV/TBEV inactivated vaccines—based on WNV strain SHUA-3 and TBEV strain Sofjin against a wide range of WNV and TBEV strains—were compared in an *in vitro* neutralization assay, as well as in BALB/c mice *in vivo*. The mono vaccines showed a lack of cross-immunogenicity and protection, whereas the combined vaccine was immunogenic against five WNV strains of lineages 1 and 2 and five TBEV strains belonging to different virus subtypes. In the mouse model, the mono-WNV vaccine was effective against the three most pathogenic strains of WNV used in this work, while the combined WNV/TBEV vaccine was effective against both WNV and TBEV. Our work shows promise for the further development of a combined vaccine against WNF and TBE.

## 1 Introduction

West Nile virus (WNV) is an emerging worldwide neurotropic orthoflavivirus transmitted by mosquitoes. Other orthoflaviviruses that are pathogenic for humans include mosquito-borne viruses, such as *Orthoflavivirus dengue (*Dengue virus), *Orthoflavivirus zikaense* (Zika virus), *Orthoflavivirus japonicum* (Japanese encephalitis virus (JEV)), *Orthoflavivirus louisense* (St Louis virus), and tick-borne viruses, such as *Orthoflavivirus encephalitidis* (tick-borne encephalitis virus (TBEV)), *Orthoflavivirus powassanense* (Powassan virus (POWV)), and *Orthoflavivirus omskense* (Omsk haemorrhagic fever virus (OHFV)) (1, 2). The orthoflaviviruses genome is approximately 11,000 kb with one open reading frame, which encodes three structural (E, prM, and C) and seven non-structural proteins (NS1, NS2a, NS2b, NS3, NS4a, NS4b, and NS5). Furthermore, mature, enveloped orthoflavivirus particles are about 50 nm in size. The surface of orthoflaviviruses is covered with 90 anti-parallel homodimers of the E structural protein, which is the main target of the antibodies produced during infection (3).

WNV’s main vector is the *Culex* mosquito; however, as numerous studies have demonstrated, other genus can also serve this function (4, 5). The circulation of this virus is affecting multiple continents including Africa, Eurasia, the Americas, and Australia (6). Annual WNV-related morbidity depends on a combination of factors such as spring rainfall, summer temperatures, fires, etc. Of particular concern is the northwards spread of the primary vector of WNV as annual temperatures rise, a phenomenon that has the potential to precipitate new outbreaks (7).

There are currently nine genetic WNV lineages. Lineage 1 is divided into lineage 1a, which is pathogenic for humans and comprises isolates from Africa (8), Europe, the Middle East, Russia, and the Americas (6, 9); lineage 1b (Kunjin virus), which is distributed in Australia (10); and lineage 1c (now reassigned to Lin 5) isolates only found in India (11). Lineage 2, which is also pathogenic to humans, circulated in Africa until 2004. Thereafter, it spread to Eastern and Central Europe and Russia (12). Lineage 3 includes some isolates from Austria and the Czech Republic (13, 14), while lineage 4 has been isolated and reported from Russia (15). Lineage 6 is based on only a small gene fragment, has been described from Spain and lineages 7 (Koutango virus) and 8 have been isolated in the Somalia and Senegal respectively (16, 17). Putative lineage 9 (or line 4c) was identified in *Uranotaenia unguiculata* mosquitoes in Austria (18).

WNV lineage 1 and 2, which are responsible for recent outbreaks, are the most pathogenic for humans (19). However, strains within each lineage can be either highly or slightly pathogenic (20).

Repeated outbreaks of West Nile fever (WNF) caused by WNV occurred in Israel in the 1950s (21). Thereafter, sporadic human cases occurred in the Europe, Western Asia and the Middle East until the year 1990 (22). Nevertheless, in 1998, there was an increase in the number of WNF cases from domestic geese, and migratory and local bird populations were associated with reported outbreaks in Romania in 1996 and in southern Russia in 1999, and Israel, in 2000 (23). A major WNF outbreak occurred in the United States from 1999 to 2004. During this period, the virus spread rapidly across the country, resulting in more than 7,000 cases of neuroinvasive WNV disease caused by lineage 1 (24). At the same time, there was an outbreak in the Volgograd region of Russia, where about 1,000 cases were reported (25). In addition, a rise in disease incidence was observed in Russia in 2010, 2012 and 2019 (26). In European countries, outbreaks were observed in 2010 (in Greece) and 2018 (predominantly in Italy) (27, 28). It should be noted that, since 2004, both lineages 1 and 2 of WNV have co-circulated in Europe and Russia (26, 29, 30).

Approximately 80% of WNF cases are asymptomatic, while the remaining 20% are cases that often present with a febrile illness. However, disease develops in less than 1% of cases, with severe CNS lesions manifesting as multiple syndromes including meningitis, encephalitis and poliomyelitis (31, 32). At the highest risk of severe forms are elderly people, and those with comorbidities, those who have undergone immunosuppressive therapy, and those who have a single-nucleotide polymorphisms in several genes (33–36).

A separate problem in adequately estimating WNF case numbers is the presence of the serological cross-reactivity with other orthoflaviviruses, in particular with TBEV. Given the symptomatic similarity of these diseases, studies demonstrate that ELISA kits, which are most often used as the primary diagnostic tool for West Nile fever and tick-borne encephalitis (TBE), do not always facilitate correct diagnosis due to their low specificity (37–39). The co-circulation of these orthoflaviviruses in the same area, as well as active tourism, complicates the situation (40, 41). Aggregate epizootic and epidemiological studies show the sympatry zones of these two viruses in Europe, including Bulgaria (42), Spain (43), Slovakia (40), Germany (44), France (45), Poland (46), Hungary (47, 48), and Slovenia (49), as well as certain regions in Russia (50). However, unlike WNV, the inactivated TBE vaccines are licensed and widely used in Europe and Russia (51–55). They have shown high immunogenicity, efficacy and good protection against the various TBEV strains circulating in different territories (55–60). A new adjuvant-free inactivated TBE vaccine, cultivated in a Vero cell culture, was also developed (61).

There is currently no licensed emergency (62) or specific prophylaxis against WNF for humans (63, 64). Due to the fact that horses and some birds, like humans, are also a dead-end hosts for WNV, a few veterinary vaccines based on the whole inactivated virus and a live chimeric virus combining prM/E WNV and a canarypox main chain, have been used and show good protection for animals (65, 66). The development of a vaccine against WNF for humans is underway; developers are employing diverse platforms and are currently in phase 1 or 2 of clinical trials (63). We have recently developed an inactivated whole-virion vaccine against WNF (67). A significant challenge in developing orthoflavivirus vaccines is the potential for antibody-dependent enhancement (ADE) of infection, which is characterized by a more severe disease course in humans with pre-existing non-neutralizing antibodies or neutralizing antibodies in the sub-neutralizing concentrations. Nevertheless, the epidemiological and experimental evidences of ADE has only been demonstrated only for dengue and Zika viruses, predominantly against mosquito-borne orthoflaviviruses. For tick-borne orthoflaviviruses, there is no strong evidence in this regard (68, 69). The presence of two orthoflaviviruses in one area makes developing a combined vaccine against TBEV and WNV an urgent task.

Here, we report an *in vivo* study of the immunogenicity and protection efficacy of both WNV alone and combined TBEV/WNV vaccines, based on inactivated virions produced in Vero cell culture against TBEV and WNV strains. The mono-WNV vaccine candidate showed immunogenicity against a wide range of WNV strains belonging to 1 and 2 lineages, as well as high protection during experimental infection. The mixed TBEV/WNV vaccine showed similar immunogenicity results against different WNV and TBEV strains, whereas the monovaccines did not offer cross-protection. The findings of the combined vaccine’s *in vivo* protection assessment demonstrated favorable outcomes against both viruses.

## 2 Materials and methods

### 2.1 Animals

Inbred BALB/c mice (State Institution Scientific Center of Biotechnology, branch “Stolbovaya”, Moscow, Russia) with a weight of 10–12 grams were used in this study. The animals were kept and treated in accordance with the international recommendations for the treatment of laboratory animals (CIOMS recommendations, 1985, the Directive 2010/63/EU, and Appendix A to the European Convention ETS No. 123). The bioethics committee of Chumakov FSC R&D IBP RAS (Institute of Poliomyelitis) (protocol #13032023 from 15 March 2023) approved all experimental procedures performed on animals.

### 2.2 Cells and viruses

The Vero cell line was cultured in DMEM (Chumakov FSC R&D IBP RAS (Institute of Poliomyelitis), Moscow, Russia) supplemented with 10% FBS (FBS, Invitrogen, Waltham, MA, USA) and penicillin/streptomycin (Paneco, Russia) at 37°C with a 5% CO2 incubator. The porcine embryo kidney (PEK) cell line was maintained at 37°C in medium 199 with Hanks’ balanced salt solution and Earle’s balanced salt solutions (2:1, v:v, (Chumakov FSC R&D IBP RAS (Institute of Poliomyelitis), Moscow, Russia)), supplemented with 5% FBS (Paneco, Moscow, Russia).

The viruses used in these study and there passage history are described in **Table 1**. The viruses were stored as aliquots of culture fluid or 10% brain suspensions in saline solution (Chumakov FSC R&D IBP RAS (Institute of Poliomyelitis), Moscow, Russia) at -80°C until use. WNV strains SHUA-1 and SHUA-3 were isolated from the serum and saliva samples, respectively, of the patient diagnosed with WNF (67). No nucleotide substitutions were observed in the structural part of the genome of these strains. Strain SHUA-3 was utilized in the preparation of the vaccine, while strain SHUA-1 was employed in the virus challenge experiments.

**Table 1:** TBEV and WNV strains and other orthoflaviviruses used in the study.

Virus	Subtype or lineage	Virus strain	Region and year of isolation	Source of isolation	Passage history	GenBank accession number
TBEV	Far Eastern	Sofjin-Chumakov	Primorsky Krai, 1937	Brain of deceased TBE patient	M_x_	KC806252
	European	256	Belarus, 1940	*I. ricinus*	M_x_M_5_P_2_	AF091014
	Siberian	EK-328	Estonia, 1972	*I. persulcatus*		DQ486861
	Baikalian-1	178-79	Irkutsk Region, Russia, 1979	*I. persulcatus*	M_x_M_1_	EF469661
	Baikalian-2	886/84	Irkutsk Region, Russia, 1984	Brain of gray-sided vole	M_x_M_1_	EF469662
WNV	2	SHUA-1	Moscow region, Russia, 2021	Blood of deceased WNV patient	V_4_	PQ679039
	2	SHUA-3	Moscow region, Russia, 2021	Saliva of deceased WNV patient	C6/36_1_M_1_V_3_	PX444460
	2	Strix nebulosa-12	Moscow, Russia, 2021	Heart of Strix nebulosa	M_1_V_3_	OP868929
	2	B-958	Unknown, before 1980	Unknown	M_x_M_1_V_1_	OQ673269
	2	Asio_otus/RUS/14/2021	Moscow, Russia, 2021	Brain of Asio otus	M_1_V_2_	PX444462
	1	HP-90	Astrakhan Region, 1963	*Hyalomma marginatum*, preimaginal stage	M_x_M_1_P_1_V_1_	PX444461
POWV		Pow-24	Primorsky Krai, Russia, 1976	*I. persulcatus ticks*	M_4_V_1_P_1_	MG652438
OHFV		Nikitina	Omsk region, Russia, 1948	Blood of a patient with OHF	M_x_M_3_P_1_V_1_ P_1_	GU290187
JEV		Jagar	Unknown, before 1975	Unknown	M_x_M_3_P_2_	AF069076.1
YFV		17D	Attenuated in 1937, during multiple passages of the Asibi strain	Blood of a patient with YF	_x_V_2_	DQ100292.1

*M—mouse brain passage (Mx—unidentified number of early passages before the virus was obtained by the laboratory); P—passage in PEK cell culture, V—passage in Vero cell culture, C6/36—passage in C6/36 *Aedes albopictus* cell.

### 2.3 Preparation of mono-WNV and combined WNV/TBEV vaccine candidates

The mono-WNV and combined WNV/TBEV vaccine candidates were prepared based on WNV SHUA-3 and TBEV Sofjin strains (Genbank KC806252), as described previously (61, 67). In brief, viruses were reproduced separately in a continuous Vero cell line. To inactivate the virus, 0.02% formaldehyde was added to the virus-containing cell culture fluid (VCF). The inactivated VCF (iWNV-VCF and iTBEV-VCF, respectively) was used to prepare mono-WNV and -TBEV concentrates. We performed clarification filtration to remove cell debris. Then iWNV and iTBEV concentrates were prepared by tangential ultrafiltration using a 300K-rated Pellicon 2 Biomax cassette membrane (Millipore, USA). The resulting concentrates were stored briefly at 2-8°C or long-term at -60 to -70°C. Both inactivated concentrates were further purified by gel filtration using Sepharose 6 FF as the sorbent (Cytiva, Marlborough, MA, USA).

Sample iWNV and iTBEV antigen concentration was estimated via a quantitative analysis of E protein content. The analysis was conducted using the ELISA Bioskin-WNV AG kit (Bioservice, Moscow, Russia) and ELISA VectoTBE-antigen kit (Vector-Best, Novosibirsk, Russia), respectively. The built-in of Thermo Scientific SkanIt PC software was used for calculations.

The final protein E concentration for the mono-WNV vaccine candidate was 3.6 µg per mL. Appropriate volumes of the both concentrates were mixed to achieve a final concentration of 3.6 μg per mL of protein E for each of iWNV and iTBEV components of the combined vaccine candidate.

### 2.4 Vaccination and virus injection

In all vaccination experiments, 8-week-old BALB/c mice were used. Animals were immunized intramuscularly at two-week intervals with 50 μl per mouse (0.1 of the human dose as estimated for EverVac vaccine (61)) into the hind thigh muscle with the mono and combined WNV/TBEV vaccine candidates. In comparative protection experiments, aluminium hydroxide (Al(OH)_3_, SPI Pharma, France) was used as an adjuvant. Al(OH)_3_ and both concentrates were mixed to achieve a final concentration of 0.8 mg/ml Al(OH)_3_, with the final concentration of the iWNV and iTBEV components remaining the same as previously described. The protein E antigen concentration for the monovaccines against TBE and WNF was 180 ng per mouse; for the combined vaccine, this was 180 ng each of TBEV and WNV antigens. Blood for immunogenicity study of the developed vaccine was taken by decapitation a total of two weeks after the last immunization. A virus challenge was also carried out at this stage at s dose 100LD_50_ for all studied viruses. The number of mice is described in the Results section for each experiment separately.

### 2.5 Analyzing the presence of WNV and TBEV RNA in mouse brain by RT-PCR

RNA extraction was performed using TRI Reagent LS (Sigma-Aldrich, St. Louis, MO, USA) according to the manufacturer’s instructions. M-MLV (Eurogen, Moscow, Russia) and WNRT-R (CGGTWYTGAGGGCTTACRTGG) for WNV or TBE/Pow3’ (5’-AGCGGGTGTTTTTCCGAGTC-3’) primers were used for the reverse transcription. PT-PCR was performed on a DNA Engine Analyzer (BioRad, Hercules, CA, USA) utilizing the RT-qPCR kit (Syntol, Russia), in accordance with the manufacturer’s instructions, using primers WNRT-R, WNRT-F (CGGAAGTYGRGTAKACGGTGCTG) and probe-WNV ((FAM)-WCCCCAGGWGGACTG-(BHQ1)) as a probe for WNV and F-TBE (5’-GGGCGGTTCTTGTTCTCC-3’), R-TBE (5’-ACACATCACCTCCTTGTCAGACT-3’) and TBE-probe ((FAM)- TGAGCCACCATCACCCAGACACA-(BHQ1)) as a probe for TBEV. The Sabin I poliovirus strain was used as the internal control, as described previously (70).

### 2.6 Virus titration in the cell cultures and 50% Plaque Reduction Neutralization Test (PRNT_50_)

As described earlier, TBEV strains virus titers were determined on the PEK cell culture in 24-well plates (71). Those of the WNV strains were established on the Vero cell culture in 24-well, in a process identical to that of the PEK cell methodology, using methylcellulose coating.

In 24-well plates, PRNT_50_ was performed on PEK cell monolayers for TBEV and on Vero cell monolayers for WNV. For the PRNT_50_ assay, mouse blood samples were collected by BALB/c mouse decapitation. Sera were obtained by centrifugation on 1000g for 30 min and stored in aliquots at −20°C. The details of the PRNT_50_ are described elsewhere (71).

Every experiment included controls, i.e., negative and positive murine sera with known antibody titers. The neutralization antibody (NAb) titers were calculated according to the modified Reed and Muench method (72).

### 2.7 Virus titration in mice

For the quantification of a 50% lethal dose (LD50) of TBEV and WNV, 8-week-old BALB/c mice in groups of five were injected intraperitoneally (i/p) with 300 μL of 10-fold dilutions of the virus in saline solution (Chumakov FSC R&D IBP RAS (Institute of Poliomyelitis), Moscow, Russia) and observed for clinical symptoms and mortality for 21 days (59). Ruffled fur, hunched posture, limb paresis and paralysis, and body weight loss were considered disease symptoms. The lethal dose of virus resulting in 50% mortality (expressed in log LD50/ml) was calculated according to the Kerber method (73).

### 2.8 WNV strains genome sequencing

To obtain the complete genome of WNV strains (Asio_otus/RUS/14/2021, HP-90), we used the specific primers described previously (67). The PCR products obtained were analyzed in an agarose gel, and bands of target length were extracted. The bands were purified using the QIAquick Gel Extraction Kit (QIAGEN, Hilden, Germany) and sequenced on the Applied Biosystems 3500 Genetic Analyzer (Waltham, MA, USA) using the BigDye Terminator v3.1 Cycle Sequencing Kit (Thermo Fisher Scientific, Vilnius, Lithuania). The resulting sequences were analyzed using Lasergene® SeqMan Pro software version 7.0.0 (DNASTAR Inc., Madison, WI, USA).

### 2.9 Phylogenetic analysis

RNA sequences of viruses from this study and some other strains of WNV and TBEV were used in the phylogenetic analysis. The nucleotide sequences of the complete fragment of protein E were aligned using ClustalW. Phylogenetic analysis was conducted using the maximum likelihood method based on the General Time Reversible model in MEGA X with 1000 bootstrap replications (74, 75).

### 2.10 Statistical analysis

Statistical analyses were performed using GraphPad Prism 9 (GraphPad Software Inc., San Diego CA, USA). For the NAb and virus titers comparisons, the Mann–Whitney U-test was used. Survival curves were analyzed by Kaplan–Meier curves using the log rank test with a Bonferroni correction for multiple comparisons. Differences in morbidity and mortality were assessed using Fisher’s exact test. Median survival time (MST) and incubation period (IP) are presented as median and range.

## 3 Results

### 3.1 *In vivo* immunogenicity and protectivity of mono-WNV vaccine against WNV strains with different virulences

**Figure 1:**
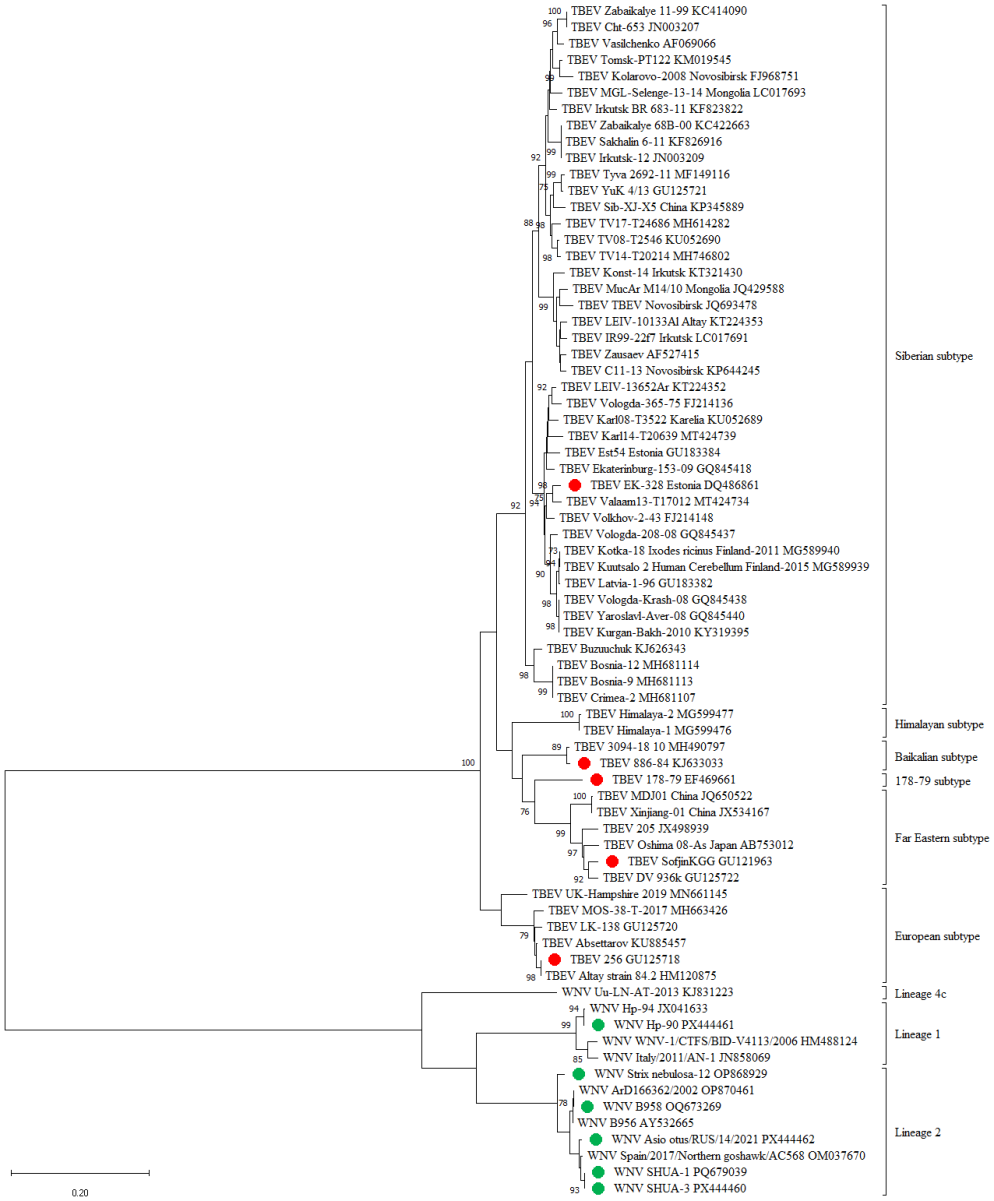
A phylogenetic tree of WNV and TBEV isolates, constructed using the maximum likelihood method using the genome region encoding protein E. The strains used in this work are highlighted in green.

It is known that WNV strains differ in terms of their propensity to induce lethal infections in both avian and mammalian hosts (76–81). Some previously characterized mutations in the genome are known to be responsible for virulence and interferon signaling, in particular in the glycosylation sites of surface protein E and in some positions in non-structural proteins (81–83). In the present study, we utilized five WNV strains belonging to lineages 1 and 2, which are the most pathogenic for humans and have been recently circulating in Europe and Russia (**Table 2**, **Figure 1**). The strains varied in virulence, with the most virulent strains in BALB/c mice being SHUA-1 and Strix nebulosa-12 (**Table 2**). Therefore, the immunogenicity of the WNV monovaccine was tested against all studied strains, and its protectiveness against the most pathogenic strains.

**Table 2:** Virulence of studied WNV strains in BALB/c mice.

Strain	Lineage	log_10_PFU/ml	log_10_LD_50_/ml^1^
SHUA-1	2	7.6±0.2^2^	6.2±0.5^3^
Strix nebulosa-12	2	7.6±0.1	4.4±0.5
Asio_otus/RUS/14/2021	2	7.4±0.1	2.6±0.5
B-958	2	6.9±0.1	<2.2±0.5
HP-90	1	7.7±0.1	2.2±0.5

^1^determined by intraperitoneal infection.

^2^Intervals mean SD.

^3^Intervals determined in a separate experiment.

**Figure 2:**
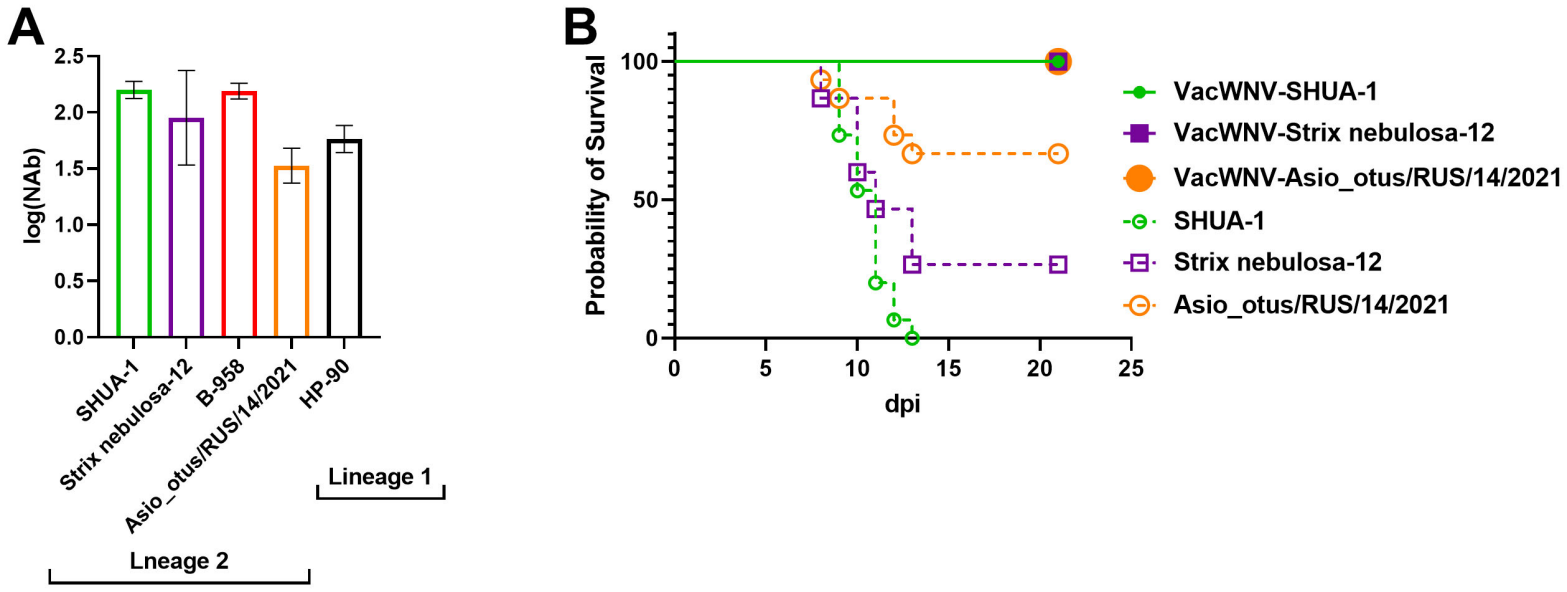
Immunogenicity and protectivity of mono-WNV vaccine. **(A)** Neutralizing antibody titers in pooled serum (N = 3) of BALB/c mice twice immunized with mono-WNV vaccine against five WNV strains (SHUA-1, Strix Nebulosa 12, B-958, Asio_otus/RUS/14/2021, and HP-90). **(B)** Survival probability of BALB/c mice twice immunized with mono-WNV vaccine (straight line) and non-vaccinated (dashed line) infected with 100 LD_50_ SHUA-1 (green circles), 100 LD_50_ Strix nebulosa-12 (violet squares) and 100 LD_50_ Asio_otus/RUS/14/2021 (orange circles) WNV strains (N = 15 for each group of mice).

Immunogenicity was tested in a separate experiment. Mouse sera were collected two weeks after double immunization with inactivated WNV monovaccine, based on the SHUA-3 strain (67), and pooled. NAbs titers against all tested strains were greater than 1:10, including the HP-90 strain from a different lineage, when compared to the vaccine strain (**Figure 2**). The vaccine demonstrated 100% protection against WNV at a dose of 100 LD_50_ for each virus strain SHUA-1, Strix nebulosa 12 and Asio otus/RUS/14/2021, which were the most pathogenic in BALB/c mice.

### 3.2 Cross-protectivity of TBE and WNV vaccines cultivated in Vero cell culture

**Figure 3:**
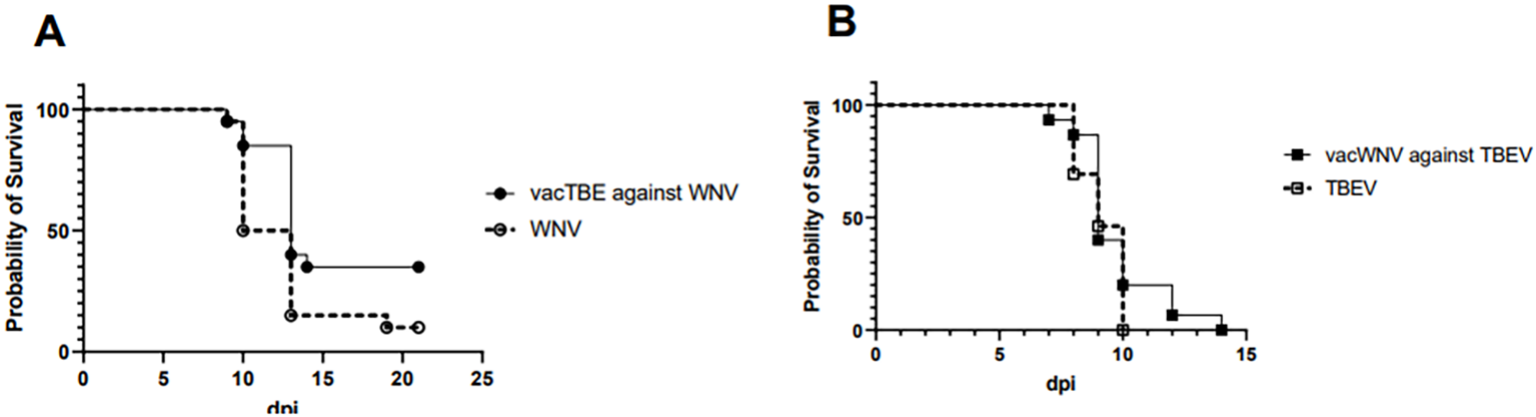
Survival probability of mice previously twice immunized with mono-TBE and -WNV vaccines. **(A)** Survival curves of BALB/c mice vaccinated with TBE vaccine (straight line) and non-vaccinated (dashed line) counterparts infected with 100 LD_50_ of WNV strain SHUA-1 (circles). **(B)** Survival curves of BALB/c mice vaccinated with WNV vaccine (straight line) and non-vaccinated (dashed line) counterparts infected with 100 LD_50_ TBEV strain Sofjin (squares) (N = 10 for each group of mice).

A study of mouse sera obtained after double vaccination with TBE (Tick-E-Vac, manufactured by the Chumakov Federal Scientific Center, Russia) or WNV monovalent vaccines cultured on Vero cells without adjuvant, in the PRNT_50_ against TBEV and WNV strains used in the vaccine (67) showed an absence of cross-reactive NAbs. In experiments on BALB/c mice testing for vaccines cross-protectivity upon infection with WNV strain SHUA-1 and TBEV strain Sofjin at a dose of 100 LD_50_, a lack of any protection was also evident (**Figure 3**).

### 3.3 Seroconversion and immunogenicity of the combined WNV/TBEV vaccine

**Figure 4:**
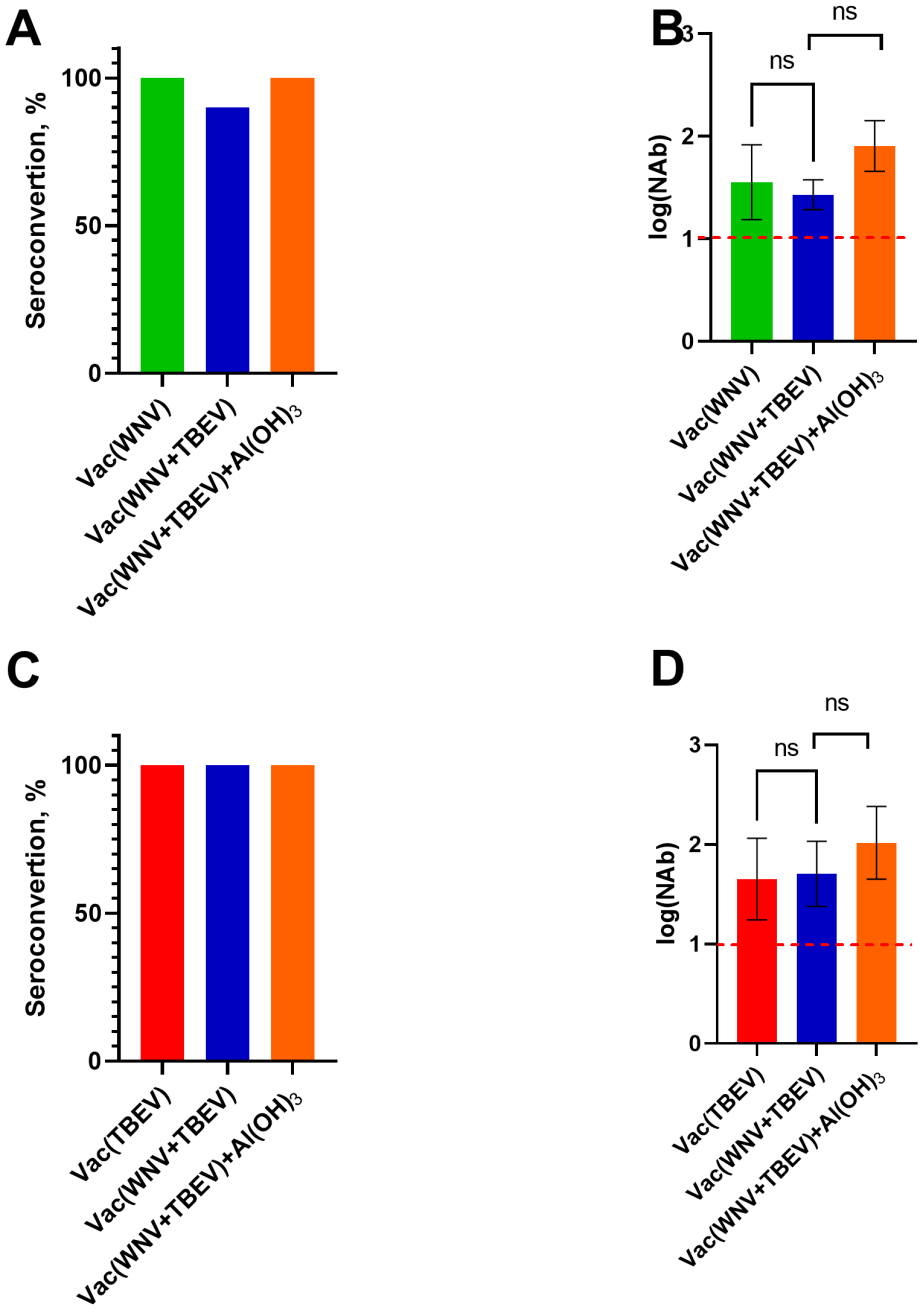
Seroconversion and immunogenicity of mono- and combined TBEV/WNV vaccines. **(A)** Seroconversion level of mono-WNV (green) or combined WNV/TBEV inactivated vaccines with (orange) and without Al(OH)_3_ (blue) against WNV strain SHUA-1 (N = 10). **(B)** NAb titers in the individual sera of BALB/c mice immunized with mono-WNV (green) or combined WNV/TBEV inactivated vaccines with (orange) and without Al(OH)_3_ (blue) against WNV strain SHUA-1 (N = 10). **(C)** Seroconversion level of mono-TBE (red) or combined WNV/TBEV inactivated vaccines with (orange) and without Al(OH)_3_ (blue) against TBEV strain Sofjin (N = 10). **(D)** NAb titers in the individual sera of BALB/c mice immunized with mono-TBE (red) or combined WNV/TBEV inactivated vaccines with (orange) and without Al(OH)_3_ (blue) against TBEV strain Sofjin (N = 10). The red dotted line indicates the protective titer of NAbs.

The combined vaccine was derived from purified inactivated whole virions of WNV and TBEV grown in Vero cells with an antigen (TBEV/WNV E protein) ratio of 180/180 ng per mouse with or without adjuvant Al(OH)_3_. The Al(OH)_3_ was chosen as an adjuvant because it is used in a licensed vaccine against TBEV, and has been shown to be both safe and effective (84). In the neutralization test, we first compared the seroconversion and immunogenicity of WNV and TBEV mono- and combined vaccines against the virus strain used in the vaccine. For the mono-WNV and combined WNV/TBEV vaccine and WNV/TBEV vaccine with Al(OH)_3_, seroconversion against the WNV strain SHUA-1 was 100, 90% and 100% respectively. For mono-TBEV and combined WNV/TBEV vaccines with or without Al(OH)_3_, seroconversion against the TBEV strain Sofjin was 100% for all (**Figure 4**). Also in the neutralization test, the immunogenicity of the monovalent and combined vaccines against WNV and TBEV did not differ and was greater than the expected protective titer (>1). No cross-protective antibodies were observed in the sera of mice after double immunization with the monovalent vaccines, either with the WNV vaccine against TBEV strain Sofjin or when immunized with TBE vaccine against WNV strain SHUA-1 (data not shown).

### 3.4 Protectivity of the combined WNV/TBEV vaccine against WNV and TBEV *in vivo*

**Figure 5:**
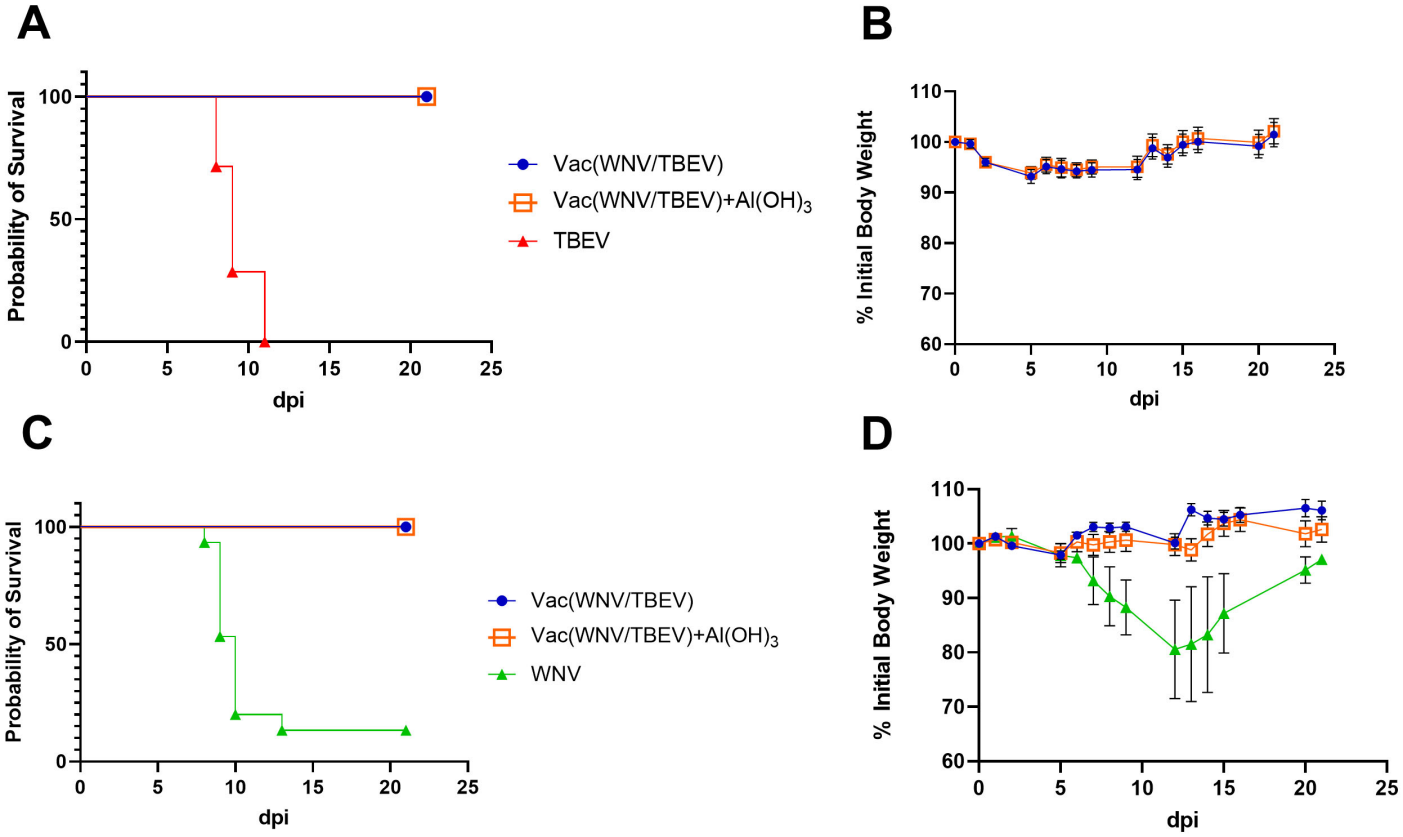
Survival probability **(A, C)** and initial body weight percentage **(B, D)** of mice twice vaccinated with combined WNV/TBEV vaccine, with (orange squares) or without (blue circles) Al(OH)_3_, and infected with TBEV strain Sofjin (**A**, red triangles) or WNV strain SHUA-1 (**C, D**, green triangles) (N = 15 for each group of mice). Weight curves are presented only for surviving mice.

The protective efficacy of the combined WNV/TBEV vaccine was evaluated in BALB/c mice against WNV strain SHUA-1 and TBEV strain Sofjin at a dose of 100 LD_50_. The immunogenicity data correlated with the protection data; the combined vaccine was 100% efficacious against the viruses tested and protecting against both morbidity and mortality (**Figure 5**). The absence of adjuvant (Al(OH)_3_) had no effect on vaccine efficacy.

Unvaccinated control animals that had been infected with WNV strain SHUA-1 and survived (2/15) exhibited signs of disease; the presence of viral RNA in the brain of one of these survivors 36 days after infection. In the group of mice that were vaccinated with the WNV/TBEV combined vaccine, no mild signs of disease were observed (**Figure 5D**, **Table 3**). In groups of animals vaccinated with the combined WNV/TBEV vaccine, viral RNA was detected in only one mouse infected with TBEV 36 days after infection (**Table 3**).

**Table 3:** Effectiveness of WNV/TBEV combine vaccine candidate against WNV strain SHUA-1 and TBEV strain Sofjin following double immunization of mice.

Group №	Combined WNV/TBEV Vaccine	Inoculated virus	Number of animals, N	Median survival time median	Incubation period median	% of survival mice	% of mice without disease symptoms	% of mice without virus RNA in brains^*^
1	+	TBEV	15	–	–	100	100	93
2	+	WNV		–	–	100	100	100
3	–	TBEV		9 [8; 11]	8 [5; 9]	0	0	0
4	–	WNV		9 [8; 13]	8 [7; 9]	13	0	7

^*^Brains were collected 36 dpi. It was taken into account that all dead mice had RNA in the brain.

### 3.5 Immunogenicity of the combined WNV/TBEV inactivated vaccine against a wide range of TBEV and WNV strains

**Figure 6:**
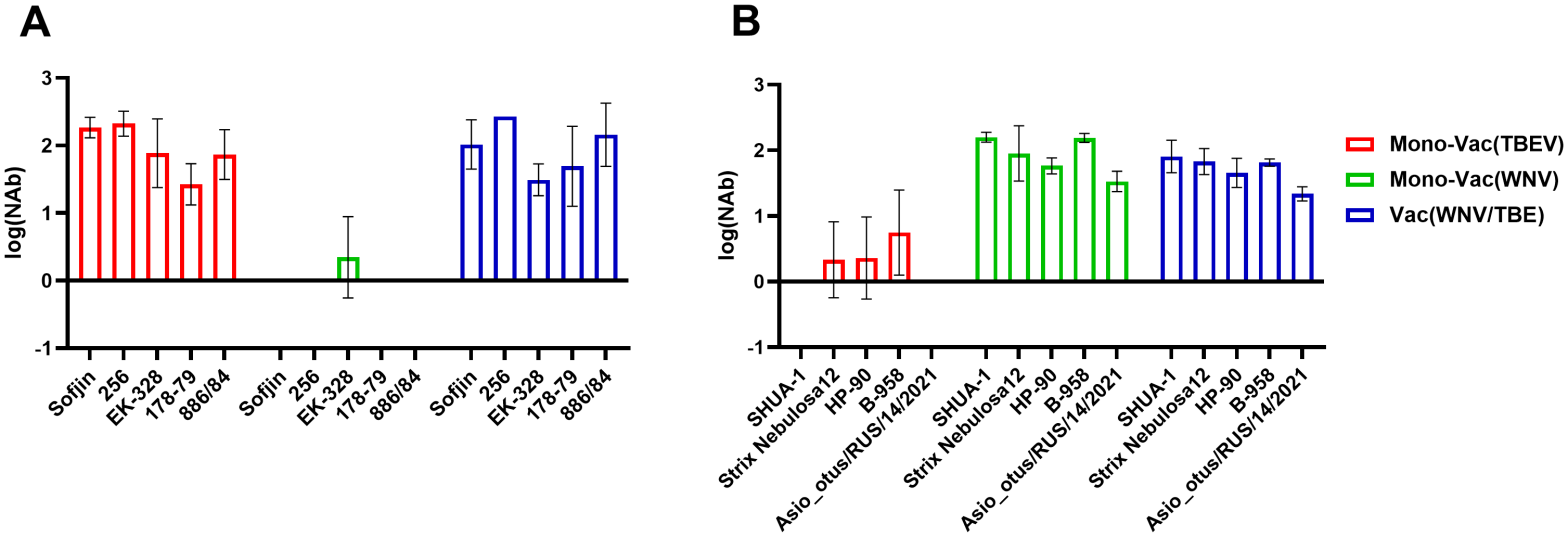
NAb titers in the pools of sera (N = 3) of BALB/c mice immunized with mono-TBEV (Mono-Vac(TBEV) group, red), mono-WNV (Mono-Vac(WNV) group, green) and combined WNV/TBEV vaccines (Vac (WNV/TBEV), blue) against different TBEV **(A)** and WNV **(B)** strains.

In view of the fact that all TBEV subtypes and several WNV lineages are currently circulating in Russia, it is imperative that the vaccine is capable of protecting against all viral strains. Sera of mice twice immunized with mono-TBEV or WNV and combined WNV/TBEV vaccines were tested against TBEV strains belonging to five different subtypes and against WNV strains belonging to two different lineages. The monovalent vaccines demonstrated minimal cross-neutralizing antibodies against some strains of heterologous viruses and NAb titers were lower than 1log_10_. In contrast, combined vaccine generated board immunogenicity to all tested TBEV and WNV strains. (**Figure 6**). The NAb titers to different strains did not differ between mono- and combined TBEV or WNV and WNV/TBEV vaccines.

### 3.6 Seroconversion and immunogenicity of WNV/TBEV combined vaccine against other ortoflaviviruses

**Figure 7:**
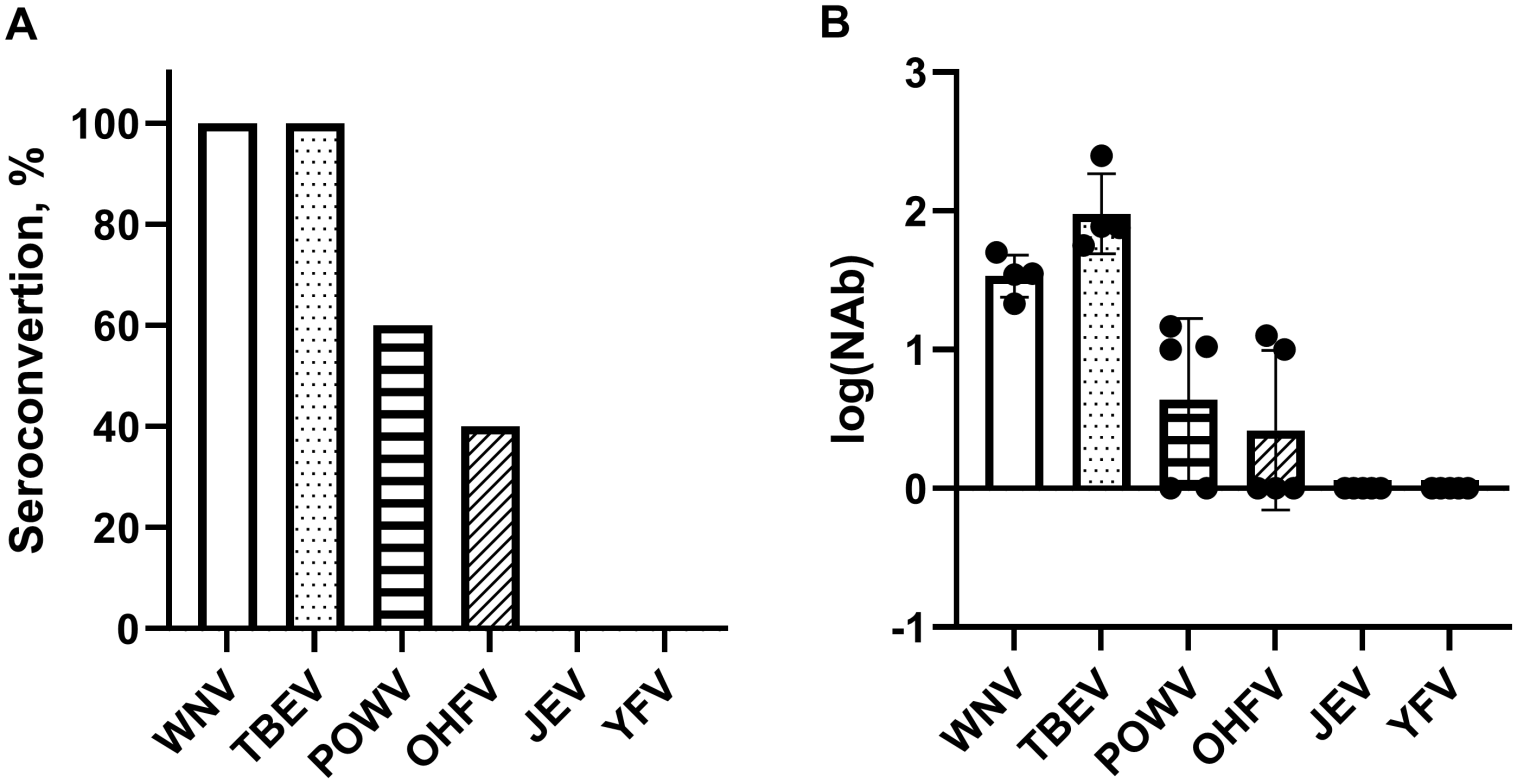
Seroconversion and immunogenicity of mono- and combined TBEV/WNV vaccines against ortoflaviviruses circulating in Russia. **(A)** Seroconversion level of combined WNV/TBEV inactivated vaccines against WNV strain SHUA-1, TBEV strain Sofjin, Powassan virus strain Pow-24, Omsk hemorrhagic fever virus strain Nikitina and Japanese encephalitis viruses strain Jagar (N = 5). **(B)** NAb titers in the individual sera of BALB/c mice immunized with combined WNV/TBEV inactivated vaccines against WNV strain SHUA-1, TBEV strain Sofjin, Powassan virus strain Pow-24, Omsk hemorrhagic fever virus strain Nikitina and Japanese encephalitis viruses strain Jagar (N = 5).

The sera of mice twice vaccinated with WNV/TBEV combined inactivated vaccine mice were tested in PRNT_50_ against OHFV, POWV, JEV and YFV. We observed a low cross-immunogenity NAbs in mouse sera against OHFV and POWV, as well as an absence of cross-immunogenity NAbs against JEV and YFV (**Figure 7**).

## 4 Discussion

WNF incidence is sporadic and outbreaks are difficult to predict because of the multifactorial nature of the virus epidemiology (83). There are currently no licensed vaccines for human WNF infection; however, it is a rising concern because of the potential for long-term neurological impairment in patients and because of the expansion of the virus into new territories (84). A human WNV vaccine is currently being actively developed using a variety of platforms (62).

In our work, we have shown that the existing inactivated TBEV vaccine does not protect against experimental WNV infection, which prompted the development of a combination vaccine against two orthoflaviviruses at once.

For mono- WNV and combined WNV/TBEV inactivated vaccines was taken from the SHUA-3 strain and tested against strains belonging to lineages 1 and 2, as it is these that are responsible for the largest disease outbreaks. The relevance of the selected strain for the vaccine against WNV is highlighted by the fact that the SHUA-3 strain was isolated from a patient with fatal West Nile fever in 2021 (67). We investigated the pathogenicity of WNV strains in mice and identified the three most pathogenic strains (Asio_otus/RUS/14/2021, SHUA-1 and Strix nebulosa-12), which were further used to study the protectiveness of the mono-WNV vaccine. It was immunogenic against all five WNV strains tested, and showed 100% protection against lethal infection and morbidity after challenge with three pathogenic strains without Al(OH)_3_.

Due to the presence of several orthoflavivirus antigens at once, we expected that the combined WNV/TBEV vaccine would show higher immunogenicity against different strains of WNV and TBEV, as well as against other orthoflaviviruses. After the administration of two doses, the TBE and WNV monovaccines did not show a cross-reactive antibody response against different TBE and WNV strains, whereas the combined vaccine was immunogenic against a broad spectrum of both virus strains. The levels of NAbs did not differ from the monovaccine immunity. When sera were tested after double immunization with the combined WNV/TBEV vaccine, NAbs levels demonstrated low titers (~1 log_10_) against Powassan and OHF viruses were detected in 2/5 sera. No NAbs against JEV and YFV were observed.

The combined WNV/TBEV vaccine has been shown to be protective against both WNV strain SHUA-3 and TBEV strain Sofjin, also used for the vaccine preparation against TBEV, in *in vivo* experiments, indicating significant potential for its further development and use.

Because of the expanding range of WNV and the difficulty in predicting outbreak locations and timings, the development of a vaccine against WNV is an important public health challenge. The use of combined vaccines has many advantages, such as reducing the number of injections and providing protection against several pathogens at once. At the same time, the use of a combined WNV/TBEV vaccine would be relevant for use in areas where there are already viral co-habitats, or where there is a risk them in the future. Our study showed that both a mono-WNV and combined WNV/TBEV vaccines based on inactivated antigens had good immunogenicity and protected against both mortality and morbidity in mouse experiments.

## Data Availability

The data presented in the study are deposited in the GenBank repository, accession numbers SHUA-3 PX444460, Hp-90 PX444461, Asio_otus/RUS/14/2021 PX444462.
